# Metachronous isolated locally advanced pancreatic metastasis from chromophobe renal cell carcinoma

**DOI:** 10.1016/j.ijscr.2019.05.046

**Published:** 2019-06-05

**Authors:** Y. Ayari, S. Ben Rhouma, H. Boussaffa, B. Chelly, K. Hamza, A. Sellami, M. Jrad, Y. Nouira

**Affiliations:** a Department of Urology, La Rabta University Hospital, Tunis, Tunisia; b Department of Anatomopathology, La Rabta University Hospital, Tunis, Tunisia; c Department of Radiology, La Rabta University Hospital, Tunis, Tunisia

**Keywords:** Chromophobe renal cell carcinoma, Metachronous, Isolated, Pancreatic, Metastasis, Treatment

## Abstract

**Introduction:**

Metastasis to the pancreas is relatively uncommon occurrence. Isolated pancreatic metastasis from renal cell carcinoma (RCC) is relatively rare and it’s usually seen in clear cell renal cell carcinoma (CCRCC), but its occurrence from chromophobe renal cell carcinoma (chRCC) is extremely rare, due to its relatively low-grade metastatic potential.

**Presentation of case:**

The authors report an unusual case of metachronous isolated pancreatic metastasis, in a 65-year-old female patient two years after left nephrectomy for chRCC, diagnosed during routine surveillance imaging and confirmed after a CT-guided pancreatic biopsy.

**Discussion:**

The pancreas is an elective site for metastases from RCC, and this particularity has been reported by several studies, but only described for CCRCC. In the English literature there is only one case of pancreatic metastasis from chRCC diagnosed in an autopsy study. Surgical resection of metastasis remains the most effective treatment, particularly for pancreatic metastases from chRCC, since radiotherapy, chemotherapy, hormonal therapy, and targeted therapy have generally proved ineffective for metastatic chRCC. In case of an unresectable disease, surgical or endoscopic palliation in association with palliative chemotherapy can improve the quality of life but not survival.

**Conclusion:**

This case highlights the unique behavior of chRCC with an unusual site of metastasis, and the necessity of long-term follow-up after primary tumor removal, even if it is known for a low-grade metastatic potential and a relatively good prognosis.

## Highlights

• Isolated pancreatic metastasis from renal cell carcinoma is relatively rare and it's usually seen in clear cell renal cell carcinoma (CCRCC), but its occurrence from chromophobe renal cell carcinoma is extremely rare.
• Pancreatic location is often diagnosed during routine surveillance imaging for the primary lesion.
• Patients in whom the pancreas is the only metastatic site and who are fit enough to undergo pancreatic surgery appear to be good candidates for the surgical treatment chemotherapy can improve the quality of life but not survival.
• It is necessary a long- term follow-up for patients treated for tumors with known low-grade metastatic potential and relatively good prognosis such as chromophobe renal cell carcinoma.

## 1 Introduction

Pancreatic metastases are rare. The most common primary source of pancreatic metastasis has been the lung followed by colon, breast, kidney, and skin. Pancreatic metastases from renal cell carcinoma (RCC) are rare and can be the only site of metastasis. It is usually described in clear cell renal cell carcinoma (CCRCC). To the best of our knowledge, we report the second case of pancreatic metastasis from chromophobe renal cell carcinoma (chRCC). Our work has been reported in line with the SCARE criteria [1].

## 2 Presentation of case

**Fig. 1:**
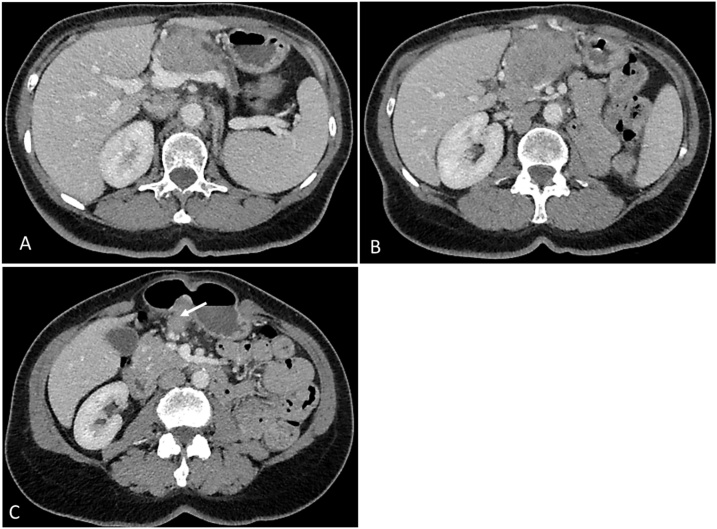
CT-scan images showing the mass of the head of the pancreas and the invasion of the superior mesenteric artery and vein (A & B). (C) Metastatic lymph node (arrow).

A 65-year-old female patient, with history of thyroidectomy for multinodular goiter, underwent a radical left nephrectomy two years ago in our department due to a renal tumor of 10 cm revealed by hematuria. Histological examination concluded to a chromophobe renal cell carcinoma (chRCC) invading the perirenal fat tissue without vascular extension nor metastases in lymph nodes. Pre-operative CT-scan showed no metastasis. The tumor was classified pT3aN0M0 according to the 2017 TNM staging system for kidney cancer. Biannual regular follow-up, showed no tumor recurrence or distant metastases. The last CT-scan, two years after the radical nephrectomy, revealed a heterogeneous mass of the pancreatic head measuring 58 × 52 × 43 mm. This mass was invading the superior mesenteric vein and artery and the celiac trunk (Fig. 1). It was associated with a suspicious lymph node of 30 mm. There were no other distant metastases.

**Fig. 2:**
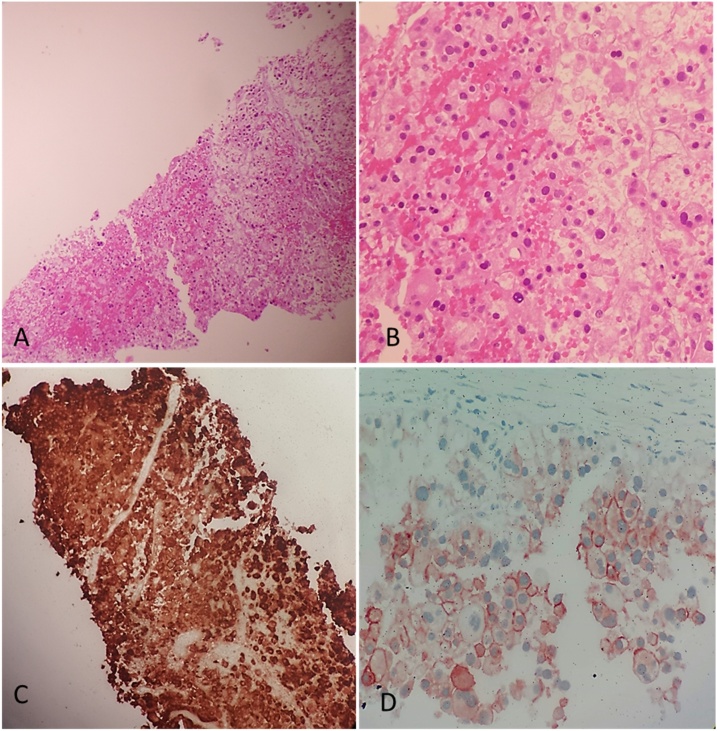
(A) HE X 20: carcinomatous proliferation with a growth pattern in islets. (B) HE X 40: large sized cells with abundant eosinophilic cytoplasm and voluminous hyperchromatic nuclei. (C) Immunohistochemical analysis showing an intense and diffuse positive staining for cytokeratin 7 (CK7). (D) Immunohistochemical analysis showing positive staining for CD117.

Physical examination was normal in particular there was no jaundice. Renal and liver laboratory tests, bilirubin, serum amylase, carcinoembryonic antigen (CEA) and CA 19-9 were normal. A CT-guided core biopsy of the lesion was performed with multiple needle passes. Microscopic examination of all biopsy specimens showed a carcinomatous proliferation with a growth pattern in islets made of large sized cells with abundant eosinophilic cytoplasm and voluminous hyperchromatic nuclei (Fig. 2A & B). Immunohistochemical staining was positive for cytokeratin 7 (CK7), CD10 and CD117. It was negative for vimentin and P504 (Fig. 2C & D). We concluded to pancreatic metastasis from chRCC.

The pancreatic metastasis was judged surgically unresectable. Thus, the patient received medical treatment with sunitinib. She is presently on the 6th cycle of oral sunitinib and is doing well with clinically asymptomatic status.

## 3 Discussion

Metastases to the pancreas are rare. Indeed, secondary neoplasms affecting the pancreas account for only 2–5% of all malignancies in the pancreas [2]. The pancreas is an elective site for metastases from RCC, and this particularity has been reported by several studies, but reported cases concerned only CCRCC [3]. Other histological types of RCC giving metastases to the pancreas are extremely rare. There was only one case of pancreatic metastasis from chromophobe renal cell carcinoma (chRCC) previously reported in the English literature and it was diagnosed in an autopsy study [4]. The most likely explanation for this unique behavior of isolated pancreatic metastases would seem to be related of a high affinity of the tumor cells to the pancreas’s parenchyma, which may explain the occurrence of metachronous late metastases [5].

Pancreatic metastases from RCC are often discovered many years after the primary diagnosis in 88% of cases and approximately 55% of patients are asymptomatic at the time of diagnosis [6]. Among patients with clinical manifestations, the most common findings are weight loss, abdominal pain, jaundice and gastrointestinal bleeding. This isolated pancreatic location is often diagnosed during routine surveillance imaging for the primary lesion [7] since patients do not present related symptoms most of the time.

Pancreatic metastasis is suspected in front of every new pancreatic lesion in patients with known history of RCC. Sometimes, it is difficult to differentiate them from adenocarcinoma of the pancreas, which are typically hypo-vascularized at the arterial time on CT-scan, unlike RCC which are hyper vascularized with hyper vascular enhancement compared with the surrounding normal pancreatic tissue [8]. Magnetic resonance imaging (MRI) describes the same hyper vascular lesions for RCC. When hyper vascular lesions are depicted on CT-scan or MRI, differentiation from a primary pancreatic endocrine tumor might be difficult hence the utility of CT guided biopsies to obtain a definitive diagnosis in some controversial cases, like in our case. However, there is a risk of hemorrhage due to the hyper vascularization of the lesions that should be taken into account. Other differential diagnoses should be considered like, metastasis of hyper vascular neoplasm, arteovenous fistulas, aneurysms of the spleen artery, and intrapancreatic accessory spleen [2].

In cases of small tumors that may be difficult to characterize with standard imaging, it is interesting to perform an endoscopic ultrasonography with fine needle biopsy, but this requires an adequate technical platform and has its own risks [9].

Chromophobe renal cell carcinoma (chRCC) is relatively rare; it accounts for approximately 2–5% of all renal malignancies, with low-grade metastatic potential and relatively good prognosis, with a high rate for both five-year recurrence-free survival (RFS) and ten-year cancer-specific survival (CSS). Yet approximately 5–10% of patients develop metastases. Thus far, there is no standard of care for metastatic chRCC [10], and actually there is limited data supporting the use of targeted therapy in metastatic chromophobe tumors [11]. Surgical resection of metastasis remains the most effective treatment, particularly for pancreatic metastases from chRCC, since radiotherapy, chemotherapy, hormonal therapy, and targeted therapy have generally proved ineffective for metastatic chRCC [10].

Patients in whom the pancreas is the only metastatic site and who are fit enough to undergo pancreatic surgery appear to be good candidates for the surgical treatment, especially if the metastases occurred after a disease free interval of more than two years [6]. Standardized pancreatic resection adapted to the location of the tumor, in terms of partial pancreaticoduodenectomy, distal pancreatectomy, and total pancreatectomy, is generally recommended for the management of isolated pancreatic metastases. Non typical local approaches, like enucleation of the tumor are confined to some exceptional cases, based on the fact that these lesions are usually well encapsuled. In cases of an unresectable neoplasm, like for our patient, surgical or endoscopic palliation in association with palliative chemotherapy can improve the quality of life but not survival.

## 4 Conclusion

Solitary localization of chRCC metastasis to the pancreas is extremely rare. They can occur many years after the removal of the primary tumor, especially if the tumor is well- differentiated. The literature on this subject is limited. There are no current recommendations for treatment. Surgery for highly selected patients would be the best therapeutic approach for this histological type of renal cell carcinoma, with long-term survival in some of them. This case highlights the necessity of long- term follow-up for patients treated for tumors with known low-grade metastatic potential and relatively good prognosis such as chRCC.

## Conflicts of interest

The authors declare that there is no conflict of in interests regarding the publication of this paper.

## Sources of funding

No source of funding.

## Ethical approval

La Rabta University Hospital ethic committee, Tunis, Tunisia.

## Consent

Written informed consent was obtained from the patient for publication of this case report and accompanying images.

## Author contribution

**Y Ayari**; concept, design, data collection, data analysis, interpretation, and writing the paper.

**S Ben Rhouma**; concept, design, data collection, data analysis, interpretation, and writing the paper.

**H Boussaffa**; data collection, data analysis, interpretation, and writing the paper.

**B Chelly**; performed the histological examination.

**K Hamza**; performed the histological examination.

**A Sellami**; data collection.

**M Jrad**; performed the CT-guided core biopsy, interpretation.

**Y Nouira**; writing the paper.

## Registration of research studies

This is no research study.

## Guarantor

Ayari Yassine.

## Provenance and peer review

Not commissioned, externally peer-reviewed.
